# Fitting additive Poisson models

**DOI:** 10.1186/1742-5573-7-4

**Published:** 2010-07-20

**Authors:** Hendriek C Boshuizen, Edith JM Feskens

**Affiliations:** 1 Department of Statistics and Mathematical Modelling, National Institute for Public Health and the Environment, PO box 1, 3720 BA Bilthoven, the Netherlands; 2 Division of Human Nutrition, Wageningen University, PO box 8129, 6700EV, Wageningen, The Netherlands

## Abstract

This paper describes how to fit an additive Poisson model using standard software. It is illustrated with SAS code, but can be similarly used for other software packages.

## 

Most epidemiological researchers analyze their data using multiplicative models, such as the Cox' Proportional Hazard Model, or logistic regression. These models assume that the risk (or the incidence, or the odds) of a disease will be multiplied by a factor when a particular exposure is present. This implies that the effect of two different exposures on the risk will be multiplicative. For instance, if a first exposure increases a baseline risk of 1 with 30% to 1.3, and another exposure doubles the risk to 2, the risk in those with both exposures will be 1.3*2 = 2,6. In some situations, however, additive models, in which risk differences from different exposures are added together, might be more appropriate. In the example given above, in an additive model the first exposure will add a risk of 0.3 to the baseline risk of 1, and the second one will add a risk of 1. Subjects having both exposures will under the additive model have a risk of 1+0.3+1 = 2.3.

Note that the term "additive models" is also used for a type of statistical models where non-parametric terms are added to the linear predictor of a model (generalized additive models [1]). This type of models is not an additive model in the sense that we use this term here.

An example of a situation where an additive model is more appropriate, is when additive effects are thought to agree more with the underlying causal mechanism [2]. For instance, when looking at numbers of deaths, it is not logical to assume that safety policies aimed at prevention of highway accidents will prevent more deaths in those with a high cardiovascular risk than in those with a low cardiovascular risk, which is what a multiplicative model would imply. Also, with continuous variables a multiplicative model implies an exponential relation between dose and response, where a linear relation might in many cases be more adequate. This is for instance the case in ecological analysis, where the exposure in the model is the frequency of exposure in a population. In that case it is logical to assume that the consequences of exposure will increase linearly with the frequency of exposure. Lastly, for public health, absolute risk differences are more important than relative differences, and additive models directly estimate those absolute differences that one is interested in.

Spiegelman and Hertzmark [3] descibed the SAS statements for fitting additive models in the case of binomial data. Here we describe how to fit an additive Poisson model in the case of counts with person time denominators. In contrast to the application Spiegelman and Hertzmark dealt with, fitting an additive Poisson model requires more changes to the statements used for fitting a multiplicative Poisson model than only changing the link = log to link = identity, which we observed that some readers of their paper have been doing. Although we use SAS GENMOD code for illustration, the principle can be applied generally in modules for generalized linear models in other statistical software packages, and to other SAS procedure that allow users to specify error distributions and the link functions, such as GAM or GLIMMIX.

In order to understand the code, we first explain the mathematical background of the statements that are needed, both for the multiplicative and the additive Poisson model. Next, we will illustrate the fitting of the additive model by running the code on an example. Lastly, we will briefly discuss the fitting problems that can occur using additive models.

## SAS statements

The SAS statements needed to fit multiplicative Poisson models are:

Proc Genmod data = datasetname;

Model ncases = var1 var2 .../link = log dist = Poisson offset = logpy;

Run;

Here ncases is the variable containing the number of cases, logpy is the variable containing the log of the person-time associated with these cases and var1 var2 ... are the independent variables in the model.

The explanation of these statements, and especially of the offset variable, is the following:

The multiplicative Poisson model for incidence is:

(1) $$I=e^{\left(\alpha +\beta_{1}\mathrm{var}1+\beta_{2}\mathrm{var}2......\right)}$$

Here I is the incidence rate, and α and β are regression coefficients.

However, in PROC GENMOD with distribution=Poisson (and in similar modules in other software packages) the dependent variable is not an incidence rates, but a Poisson variable, i.e. a count. So it does not model the incidence, but the number of cases. Therefore one first has to rewrite (1) in terms of number of cases, using:

(2) $$I=\frac{c}{py}\Leftrightarrow \log\left(I\right)=\log\left(c\right)-\log\left(py\right)$$

where c is the number of cases, and py the person time.

Taking the log on both sides of equation (1) and using (2) yields:

$$\log\left(c\right)=\log\left(py\right)+\alpha +\beta_{1}\mathrm{var}1+\beta_{2}\mathrm{var}2.....$$

This model then is fitted in PROC GENMOD, where the log on the left side of the equation is represented by link = log, and the term log(py) has to be included as an offset: an offset is data-item that is included in the model, and which can differ for each data-record. It can be seen as a variable for which the regression coefficient is constrained to be 1.

In the case of an additive Poisson model, the model to be fitted is:

(3) $$I=\frac{c}{py}=\alpha +\beta_{1}\mathrm{var}1+\beta_{2}\mathrm{var}2.....$$

A rewrite into a model for c yields in this case:

(4) $$c=py+\alpha py+\beta_{1}py\mathrm{var}1+\beta_{2}py\mathrm{var}2.....$$

In GENMOD statements the model therefore will be:

Proc Genmod data = datasetname;

Model ncases = py py*var1 py*var2 .../link = identity dist = Poisson noint;

Run;

Here the noint means that no intercept is fitted, as the intercept of model (3) is represented by the coefficient of the py-term. Similarly, in (3) is given by the regression coefficient for py*var1.

Similar statements are needed for an additive model for the SMR, replacing person-time with expected cases, as has been described before [4].

## Example

To illustrate the model, we fitted an additive Poisson model on 1993-2007 hospital admission data for coronary heart disease in the Dutch population aged 50 to 85, and related them to smoking rates. In such an ecological setting, incidence of smoking will be linearly related to the number of smokers in the population, and therefore an additive Poisson model is required to fit these data. The number of hospital admissions and population data were taken from the public database of Statistics Netherlands (statline.cbs.nl), stratified by gender and 5-year age group. Population numbers were divided by 10,000 in order to let our coefficients represent incidence per 10,000. Smoking rates (percentages) were taken from the trend publication of STIVORO (the Dutch expert centre on tobacco control) [5], and were given for the ages 50-64 and 65 and over. We also added a time trend to the model in order not to ascribe time trends to smoking effects, and entered age as categorical variable (using 5-year age categories) in order to capture any non-linear effects of age.

The SAS code used was:

proc genmod;

class age (ref = first)/param = ref;

model coronary_heart_disease = npop npop*year npop*smokeperc npop*age npop*sex/noint link = identity dist = poisson lrci dscale;

run;

We added a scale factor to accommodate the over-dispersion in these data (option dscale), and the option "lrci" that provides likelihood ratio confidence intervals, which are more reliable than Wald intervals as they do not depend on estimates of standard errors. We also parameterized age as 0/1 dummy variables (option param = ref), using the lowest age (50-54) as the reference category (option ref = first).

The SAS output of this model is shown in table 1.

**Table 1:** Analysis Of Maximum Likelihood Parameter Estimates

Parameter		DF	Estimate	Standard Error	95% Confidence Limits		Wald Chi-Square	Pr > ChiSq
Intercept		0	0.0000	0.0000	0.0000	0.0000		
npop		1	149.7511	20.5746	109.4631	190.0986	52.98	<.0001
npop*year		1	-0.9299	0.3403	-1.5973	-0.2637	7.47	0.0063
npop*smokeperc		1	0.9771	0.5512	-0.1011	2.0589	3.14	0.0763
npop*age	57	1	28.8675	3.2461	22.5631	35.2959	79.08	<.0001
npop*age	62	1	73.0960	4.2089	64.9437	81.4464	301.61	<.0001
npop*age	67	1	137.7084	8.5150	121.0384	154.4137	261.55	<.0001
npop*age	72	1	177.6408	9.0233	159.9900	195.3591	387.58	<.0001
npop*age	77	1	183.7507	9.5126	165.1688	202.4576	373.13	<.0001
npop*age	82	1	154.4523	9.9867	134.9989	174.1502	239.19	<.0001
Npop*sex		1	-135.249	5.2618	-145.558	-124.933	660.70	<.0001
Scale		0			10.1166	10.1166		

Here the regression coefficient for npop represents the baseline incidence fitted by the model, that is the incidence (per 10.000 person-years) in 1993, in a non-smoking male population aged 50-54. The coefficient of 0.977 for npop*smokeperc indicates that, according to this analysis, an increase of the percentage of smokers with 1 percent will yield approximately 1 extra hospital admission for CHD per 10.000 person-years. We would like to stress that we include this example to illustrate the use of additive regression, and therefore kept the model simple, too simple to be realistic. We therefore discourage the drawing of any subject matter conclusion from these results.

## Non convergence

A common problem with the additive Poisson model is that the model algorithm fails because for some records the estimated number of cases becomes negative. It is possible to work around this by using the programming statements of PROC GENMOD to reprogramming the fitting algorithm, changing negative fitted values into small positive values before calculating their contribution to the deviance. However, this is not recommended, as failure to fit to model generally indicates that the model does not fit the data. For instance, in epidemiology the relation between incidence and age is often non-linear. Including a linear age term in this situation automatically leads to fitted values that are too low at young age, and, given that the risks at low age are already low, they then easily become negative. Modeling age in a more realistic way (e.g. using polynomial terms or exp(age) as covariate) might yield a model that fits the data better, and does not cause errors.

Similarly, the additive model assumes that all variables add a constant amount to the incidence rate, irrespective of the baseline risk (i.e. the risk the person has because of his/her other risk factors). If in reality the extra risk is lower in those with a low "baseline" risk, then this assumption will fit too large a risk difference in those with low baseline risk, and this might also lead to expected negative risks. In this case a multiplicative model might fit the data better. If a multiplicative effect is only observed for one or two factors, while effects of other factors are additive, one might try adding interaction-terms of these factors. Alternatively, one could use an additive multiplicative hazard model [6], where part of the covariates have an additive effect on the baseline hazard, while others have a multiplicative effect.

Careful modeling, starting with fitting a simple model and extending the model gradually is recommended when fitting problems occur, in order to find the aspects of the data that causes the problem. Also, when the likelihood from subsequent iterations is still increasing at the moment the maximum number of iterations has been reached, increasing the maximum number of iterations can help. If this is not the case, using a less restrictive convergence criterion could help (either CONVH or CONVERGE, dependent on which convergence criteria is not satisfied).

In our example, we used profile likelihood confidence bounds for our parameters, as they require fewer assumptions than Wald confidence intervals. In additive models, parameters can be close to the boundary of the parameter space. In such cases, the assumption of a symmetric confidence interval implied by the Wald interval may be unrealistic. Using the profile-likelihood interval, however, is not always the remedy, as they might fail to converge in situations close to the boundary of the parameter space.

Although the fitting problems with additive models are often regarded as a drawback of these models, one can also regard them as an advantage, as it prevents fitting of models that do not fit the data, and encourages critical reflection on the way data should be modeled. Hopefully, therefore, this contribution will promote use of additive models in epidemiology, especially as absolute rate differences are often more relevant for public health then rate ratio's.

## Competing interests

The authors declare that they have no competing interests.

## Authors' contributions

HB drafted the manuscript and conceived of the method, and FE revised the manuscript for intellectual content. Both authors read and approved the final manuscript.
